# Clinical Outcomes of Wulingsan Subtraction Decoction Treatment of Postoperative Brain Edema and Fever as a Complication of Glioma Neurosurgery

**DOI:** 10.1155/2016/5078689

**Published:** 2016-02-25

**Authors:** Wei-rong Jin, Feng-e Zhang, Bao-zhong Diao, Yue-ying Zhang

**Affiliations:** ^1^Formulation Branch Department, Liaocheng People's Hospital, Liaocheng 252000, China; ^2^Emergency Department, Shandong Liaocheng People's Hospital, Liaocheng 252000, China; ^3^Institute of Basic Medicine, Shandong Academy of Medical Sciences, Jinan 250014, China

## Abstract

*Objective.* To evaluate the efficacy of Wulingsan subtraction (五苓散加减 WLSS) decoction in the treatment of postoperative brain edema and fever as a complication of glioma neurosurgery.* Methods.* This retrospective study was conducted at the Department of Neurosurgery of Liaocheng People's Hospital. Patients hospitalized between March 2011 and December 2014 were divided into three groups: Group A received WLSS oral liquid (50 mL), twice a day; Group B received an intravenous infusion of mannitol; and Group C received WLSS combined with mannitol (*n* = 30 patients per group). All patients were treated for 10 days continuously. Therapeutic efficacy was evaluated by measuring body temperature and indicators of renal function before and 3, 5, and 10 days after treatment.* Results.* Compared to the other two groups, significantly greater clinical efficacy was observed in the patients treated with mannitol (Group B; *P* < 0.05), although marked clinical efficacy was also observed over time in patients treated with WLSS (Group A). After 5 days, the quantifiable effects of the WLSS and mannitol combination group (Group C) were substantial (*P* < 0.05). The renal damage in Group B was more obvious after 5 days and 10 days.* Conclusion.* Compared with mannitol treatment alone, WLSS combined with mannitol induced a more rapid reduction in body temperature. Our findings suggest that patients should be started on mannitol for 3 days and then switched to WLSS to achieve obvious antipyretic effects and protect renal function. This method of treatment should be considered for clinical applications.

## 1. Introduction

Postoperative brain edema and fever are an important complication of glioma neurosurgery. As a conventional medicine, mannitol is commonly used to treat cerebral edema in neurosurgery. However, thrombophlebitis and kidney damage are associated with long-term use of mannitol. Wulingsan (五苓散), which was originally reported in the “Treatise on (《伤寒论》),” is a yang gas, water line dampness herb used in traditional Chinese medicine. In this study, WLSS was composed of Wulingsan (五苓散), Astragall Radix, and Rhizoma Anemarrhenae.

Glioblastoma (GBM) is the most common and the most malignant tumor of the brain [1], accounting for 35.2%–61.0% of all intracranial cancers, with an average incidence of 49.7%. At present, cerebral glioma is most commonly treated in the clinic by surgery (including total and subtotal tumor removal) with appropriate radiotherapy and chemotherapy [2]. Due to alterations in the blood-brain barrier (BBB) induced by vascular endothelial growth factor- (VEGF-) mediated angiogenesis and leading to peritumoral edema, chronic administration of steroids is usually required to minimize neurological damage. Postoperative brain edema and fever as a complication of glioma neurosurgery are some of the most common complications faced by the Department of Neurosurgery, with the incidence of fever reported to exceed 22% [3]. For almost a century [4], mannitol has been used widely used as the conventional treatment of various types of brain edema. Recently, it has become the most effective first-line drug used in the Department of Neurosurgery for the treatment of encephalopathy crisis, emergency rescue, and dehydration conditions. However, long-term application mannitol is associated with hyponatremia, reduced blood pressure, and renal damage [5]. Natural plants products are a valuable source of novel compounds that can be utilized to combat tumor cells. More than 20 aster species have been used in traditional Chinese medicine to treat snakebites, fever, cold, tonsillitis, and pneumonia [6]. In the clinic, we found good effects of Wulingsan subtraction (WLSS) plus* Astragalus* and* Rhizoma Anemarrhenae* for the treatment of brain edema and fever as a complication of glioma neurosurgery, without the toxic side-effects of mannitol.

In this study, we performed a retrospective analysis of the efficacy of decoction of WLSS and mannitol alone and in combination for the treatment of postoperative brain edema and fever as a complication of glioma neurosurgery to determine which approach is the most conducive to the treatment of such patients.

## 2. Methods and Patients

### 2.1. Study Design

This retrospective study was conducted at Liaocheng People's Hospital (Brain Branch) between March 2011 and 31 December, 2014. The protocols were approved by the hospital Ethics Committee. All patients (or the guardian of a minor) provided written informed consent before enrollment.

### 2.2. Inclusion Criteria

The inclusion criteria were as follows: patients with brain glioma confirmed by MRI; successful glioma resection; four days of postoperative axillary temperature over 37.5°C; aged 3–65 y.

### 2.3. Exclusion Criteria

The exclusion criteria were as follows: patients with fever (below 38.0°C) beginning within 3 days after surgery; patients with normal axillary temperature by day 4 after surgery; patients with fever caused by lung infection or other infections; patients with severe heart, liver, and kidney dysfunction.

### 2.4. Patients

Patients with postoperative brain edema and fever as a complication of glioma neurosurgery were recruited from Liaocheng People's Hospital Department of Neurosurgery during the period from March 2011 to December 2014. A total of 90 cases were included in our retrospective analysis.

### 2.5. WLSS Composition

WLSS composition is as follows:* Poria cocos* for Poria (18 g) (with efficacy of eliminating dampness and diuresis), Alisma families* Rhizoma Alismatis* (30 g) (with efficacy of expelling dampness to remove fever), Compositae of rhizoma atractylodis (stir-fried) (10 g) (with efficacy of drying damp and strengthening spleen),* Cinnamomum cassia* Presl* Ramulus Cinnamomi* (12 g) (with efficacy of tonifying fire and helping yang), Leguminous* Astragall Radix* (10 g) (with efficacy of swelling water and tonifying Qi), and Liliaceous* Rhizoma Anemarrhenae* (6 g) (with efficacy of clearing heat-fire and Fluiding dryness).

Above 8 herbs were heated and boiled in water twice: first, for two hours and, second, for 1.5 hours. Then follow these steps: the merger decoction, filtration, the filtrate being concentrated to about 800 mL, adding water to 1000 mL, mixing, packaging, and sterilization.

### 2.6. Groups and Treatment

According to the accepted methods of treatment, all patients were divided into three groups: WLSS group (Group A), 30 patients with a mean age of 43.21 ± 12.25 y; mannitol group (Group B), 30 patients with a mean age of 43.19 ± 10.15 y; WLSS combined with mannitol as a dehydration treatment (Group C), 30 patients with a mean age of 42.98 ± 10.11 y.

The groups were treated at the same time and for the same duration as follows: Group A received WLSS oral liquid (50 mL) twice a day (the dose was reduced appropriately for treatment of children). Patients with persistent fever (axillary temperature maintained above 38°C for three days) were retreated using WLSS 50 mL, three times a day. Group B received an intravenous infusion of mannitol using the routine method. Group C received WLSS treatment as described for Group A in addition to conventional treatment with mannitol as described for Group B. All patients received 10 days of continuous treatment (1 course). Each group of average data is comparable (*P* > 0.05).

### 2.7. Detection of Temperature and Renal Symptoms

Details of temperature, signs, tongue examination, and pulse were recorded before and after treatment in the morning with the patient in the supine position. Axillary temperature was recorded using a mercury thermometer every 5 minutes. Indicators of renal toxicity [7, 8] cystatin C (Cys-C), serum creatinine (Scr), blood urea nitrogen (BUN), and *α*-Microglobulin (*α*1-MG) were measured in routine blood tests. Measurements were made before and 3, 5, and 10 days after treatment.

### 2.8. Assessment of Treatment Efficacy [9]

The efficacy of the treatment received by the patients in each groups was categorized as follows: cure: the temperature of the patient returned to normal (36–37°C) following treatment for 10 days; marked efficacy: the temperature of the patient fell by 1.5°C but had not returned to normal following treatment for 10 days; effective: the temperature of the patient fell by 0.5–1.5°C but had not returned to normal following treatment for 10 days; invalid: the temperature of patients changed by <0.5°C following treatment for 10 days.

### 2.9. Statistical Analysis [10]

All data were analyzed by one analysis of variance, and the differences between means were established by Duncan's multiple-range test. Data represent means and standard deviations. *P* < 0.05 was considered to indicate statistical significance.

## 3. Outcomes

### 3.1. Demographic Characteristics and Baseline Measurements

This study is a retrospective study. Among the 102 eligible patients, 12 died. Among the 90 remaining eligible patients, there were no significant differences between the groups in terms of demographic characteristics and baseline body temperature without renal dysfunction (Table 1).

Different degrees of efficacy were observed among the groups after treatment for 3, 5, and 10 days. Compared to the other two groups, significantly greater clinical efficacy was observed in the patients treated with mannitol (Group B; *P* < 0.05), although marked clinical efficacy was also observed over time in patients treated with WLSS (Group A) (Table 2).

The kidney damage observed in patients following the three modes of treatment are shown in Table 3. Kidney damage was significantly minimized in Group A, and there was a significant difference among the three groups (*P* < 0.01).

## 4. Discussion

Study shows the treatment mode: begin using nontoxic kidney with mannitol WLSS 3 days after treatment and then alone use WLSS, not only to achieve the purpose of cooling, but also to protect the kidneys. A large number of studies have shown that the WLS for a variety of kidney diseases have certain curative effect. In 2008, He et al. [11] reported that Wulingsan suppressed the development of calcinosis in rats fed the high phosphorus diet supplemented with this Chinese medicine. Liu et al. [12], through anticancer agent adriamycin-induced nephrotic syndrome in rats by Wulingsan (Gorei-San) study, have found amelioration of anticancer agent adriamycin-induced nephrotic syndrome in rats by Wulingsan (Gorei-San), a blended traditional Chinese herbal medicine.

Other studies have reported [12, 13] that Wulingsan inhibits the development of renal calcium AD cool-headed disease and also has certain effect on high phosphorus diet induced mouse. Whether through suppressed the development of calcinosis mechanism to protect renal is a question worthy of further study.

Mannitol is a hypertonic dehydration agent widely used for fast, effective, and accurate relief of intracranial pressure in the clinical treatment of cerebral edema the prevention of acute renal failure and glaucoma and also to rescue clinical brain disorders, particularly those caused by a rapid increase in blood pressure. It can accelerate kidney poison and drug excretion. In recent years, studies have shown that if limited quantities of mannitol are available, it should be used to treat patients at particularly high risk of nephrotoxicity preferentially [14]. In 2014, Morgan et al. [15] reported that the nephrotoxic potential of high-dose mannitol and the prolonged use of mannitol to reduce intracranial pressure during glioma surgery can cause renal toxicity.

Although medicine and time may have reciprocal effects on intracellular calcium concentrations, the present study is the first to investigate the efficacy of WLSS subtraction decoction for the treatment of postoperative brain edema and fever following glioma surgery. Mannitol showed rapid onset clinical efficacy with renal toxicity, while WLSS showed good clinical efficacy without nephrotoxicity, although the onset of its effects was slower. Our findings suggest that, for the treatment of postoperative brain edema and fever following glioma surgery, patients should be started on mannitol for 3 days and then switched to WLSS. This method of treatment should be considered for clinical applications.

## Figures and Tables

**Table 1:** Patient grouping.

Group	Sex	Age	Temperature	Means
	(Male/female)		High/low	
A 组	12/18	43.21 ± 12.25	41.0/38.0	39.11 ± 1.32
B 组	15/15	43.19 ± 10.15	40.8/38.5	39.09 ± 0.91
C 组	14/16	42.98 ± 10.11	40.0/38.1	39.30 ± 2.01
^*∗*^ *P*		0.9966		0.8332

^*∗*^ *P* > 0.05 (among the three groups).

**Table 2:** Comparison of the three treatment groups.

Group	WLSS	Mannitol	Combination
Cases	30	30	30
3 days of significant effect (%)	22 (73.33)	28 (93.33)^*∗*^	28 (93.33)^*∗*^
5 days of significant effect (%)	28 (93.33)^*∗*^	23 (76.67)	20 (66.67)
10 days of significant effect (%)	28 (93.33)^*∗*^	28 (93.33)^*∗*^	29 (96.67)^*∗*^
10 days invalid (%)	0	0	0

^*∗*^ *P* < 0.05, in accordance with Table 1 cluster approach.

**Table 3:** Kidney damage of three administration modes.

Group	Detection index			
	Cys-C (mg/L)	SCr (*μ*mol/L)	BUN (mg/dl)	*α*l-MG (mg/L)
Group A				
Before treatment	1.89 ± 0.19	67.01 ± 2.11	28.23 ± 2.32	28.01 ± 2.56
3 d after treatment	2.69 ± 0.29	99.98 ± 10.32	42.21 ± 4.01	41.00 ± 3.99
10 d after treatment	2.31 ± 0.33	90.01 ± 8.01	33.72 ± 2.95	29.87 ± 2.67

Group B				
Before treatment	1.89 ± 0.19	67.01 ± 2.11	28.23 ± 2.32	28.01 ± 2.56
3 d after treatment	2.66 ± 0.33	100.11 ± 10.09	43.00 ± 3.88	39.97 ± 3.51
10 d after treatment	2.29 ± 0.24	90.31 ± 7.97	32.01 ± 2.77	27.74 ± 2.13

Group C				
Before treatment	1.89 ± 0.19	67.01 ± 2.11	28.23 ± 2.32	28.01 ± 2.56
3 d after treatment	1.99 ± 0.17	65.44 ± 7.16	36.96 ± 3.81	34.97 ± 2.99
10 d after treatment	1.78 ± 0.22	63.35 ± 6.66	26.84 ± 3.22	25.37 ± 2.22

(1) A compared with C	*P* ^△^ = 0.0000	*P* ^△^ = 0.0000	*P* ^△^ = 0.0000	*P* ^△^ = 0.0000
	*F* ^△^ = 84.70	*F* ^△^ = 184.53	*F* ^△^ = 28.33	*F* ^△^ = 33.58

(2) B compared with C	*P* ^▲^ = 0.0000	*P* ^▲^ = 0.0000	*P* ^▲^ = 0.0000	*P* ^▲^ = 0.0000
	*F* ^▲^ = 5.39	*F* ^▲^ = 167.14	*F* ^▲^ = 57.57	*F* ^▲^ = 36.64

	*P* ^△^ > 0.05	*P* ^△^ < 0.01;	*P* ^1^_A,C_ < 0.01;	*P* ^1^_A,C_ < 0.01;
	*P* ^▲^ < 0.01	*P* ^▲^ < 0.01	*P* ^2^_B,C_ < 0.01	*P* ^2^_B,C_ < 0.01

▲, △, 1, and 2 refer to B compared with C, A compared with C respectively.
